# Study of calcitriol anti-aging effects on human natural killer cells *in vitro*

**DOI:** 10.1080/21655979.2021.1972076

**Published:** 2021-09-21

**Authors:** Weiran Li, Xu Che, Xuemei Chen, Meiling Zhou, Xiaoping Luo, Tao Liu

**Affiliations:** a Department of Oncology Rehabilitation, Shenzhen Luohu People’s Hospital, the 3rd Affiliated Hospital of Shenzhen University, Shenzhen, China; b Medical Laboratory of Shenzhen LuoHu People’s Hospital, Shenzhen, China; c Department of Hepatobiliary & Pancreatic Surgery, National Cancer Center/National Clinical Research Center for Cancer/Cancer Hospital & Shenzhen Hospital, China; d Department of Pancreatic and Gastric Surgery, National Cancer Center/National Clinical Research Center for Cancer/Cancer Hospital, Chinese Academy of Medical Sciences and Peking Union Medical College, Beijing, China

**Keywords:** Calcitriol, nk cells, SIRT1-∆Exon8, anti-aging, oxidative senescence

## Abstract

Vitamin D is widely considered to have a regulatory effect on the immune system. Some clinical investigations have shown that the demand for vitamin D increases with age. Calcitriol is the biologically active form of vitamin D. However, its effect on human natural killer (NK) cells remains unclear. Therefore, in this study, we investigated the anti-aging and immunomodulatory effects of calcitriol on NK cells using a series of immunological methods to explore its important role in innate immunity. We found that calcitriol reversed the expression of aging-related biomarkers in NK cells and inhibited their expansion by maintaining these cells in the G1 phase, without any apoptosis and exhaustion. Calcitriol repressed the release of inflammation-related cytokines, such as interleukin-5 (IL-5), interleukin-13 (IL-13), interferon-gamma (IFN-γ), and tumor necrosis factor-alpha (TNF-α). The degranulation of NK cells was downregulated by calcitriol when these cells were co-cultured with K562 tumor cells. We also found that calcitriol upregulated the aging-related sirtuin 1- protein/kinase R-like endoplasmic reticulum kinase (SIRT1/pERK) pathway and SIRT1-deltaExon8 (SIRT1-∆Exon8) expression by activating the vitamin D receptor (VDR). Moreover, calcitriol could be a potential negative regulator of NK cell apoptosis and mitochondrial inactivation which caused by oxidative stress. Thus, calcitriol exhibits anti-aging effects on human NK cells *in vitro* by activating the SIRT1-PERK axis and resisting oxidative senescence.

## 1. Introduction

Many studies have found that aging exerts a direct influence on several diseases, such as infectious diseases, cardiovascular diseases, neurodegenerative diseases, autoimmune disorders, and cancer. Organism aging is accompanied by immunosenescence. The common features of aging mainly include inflammageing with chronic low inflammation [1], genome instability, protein expression imbalance, mitochondrial inactivation, cellular senescence, and changes in intercellular communication [2].

As the main component of innate immunity, NK cells play an important role in the anti-infection and anti-tumor effects as well as the regulation of the immune system [3]. NK cells can eliminate the target cells directly or via antibody-dependent cellular cytotoxicity (ADCC), instead of exhibiting any antigen- or antibody-specific responses [4]. NK cells are also associated with hypersensitivity reactions and autoimmune diseases [5]. When the immune system getting aged, the expression of surface activation receptors such as the natural killer group 2 member D (NKG2D) [6], killer immunoglobulin-like receptor (KIR), surface marker cluster of differentiation 57 (CD57) [7], cluster of differentiation 16 (CD16) [8] increased. These molecule could activate the killing function of NK cells, which may leads to cellular imbalance and inflammation in elder. Meanwhile, the inhibitory molecules of NK cells such as natural killer group 2 member A (NKG2A), natural killer group 2 member C (NKG2C), and natural cytotoxicity receptors (NCRs) [6] decreased. Other aging-related markers of NK cells are T cell immunoglobulin and mucin domain-containing protein 3 (TIM3) and programmed cell death-1 (PD-1) increased. The upregulation of PD-1 and TIM3 during immunosenescence may inhibit the functions of NK cells and leads to NK cell depletion [9].

Another factor leading to immunosenescence is immune-inflammation [10]. High levels of inflammatory cytokines released by NK cells have been reported to cause DNA damage, oxidative stress, and cell senescence. Moreover, the cellular imbalance of NK cells and inflammation status may result in autoimmune disease [11]. The levels of interleukin-1 (IL-1), interleukin-4 (IL-4), interleukin-6 (IL-6), interleukin-10 (IL-10), and tumor necrosis factor-alpha (TNF-α) are higher in elderly people [12]. Although the number of total immune cells decreased with aging, the NK cell population remarkably increased, which may be caused by the infiltration of inflammatory cells [13].

Vitamin D exists in two active forms in the human body as vitamin D2 (ergocalciferol) and vitamin D3 (calcitriol). The main function of vitamin D is to promote the absorption of calcium and phosphorus. Vitamin D also regulates the immune functions [14]. Although many reports have focused on its anti-infection and anti-tumor functions [15], calcitriol exhibits anti-inflammatory effects as well [16]. The vitamin D receptor (VDR) is expressed on various immune cells [17]. Decrease in the production of inflammatory cytokines contributes to the reduction in inflammatory reactions. Calcitriol is known to suppress the production of TNF-α, interleukin-1 (IL-1), interleukin-2 (IL-2), interferon-gamma (IFN-γ), and other inflammatory cytokines in B and T cells. It can also release anti-inflammatory cytokines like IL-4 and IL-10 [18] and regulate autophagy against inflammatory cell infiltration [19].

The antioxidant properties of calcitriol have been reported in depression, fatty liver disease [20], cardiovascular disease, and other inflammation-related diseases. Free radicals and reactive oxygen species (ROS) also accumulate in cells and tissues during aging. Published data have demonstrated that oxidative stress affects a variety of signaling pathways in cells [21], including the SIRT1, mitogen-activated protein kinase (MAPK), and nuclear factor-κB (NF-κB) pathways. The *SIRT1* gene is considered as a longevity gene and the suppression of this gene results in disorders related to protein expression, cell cycle, and metabolism [22]. SIRT1-ΔExon8 is a novel isoform of SIRT1, which existed in mammals by alternatively splicing. SIRT1-ΔExon8 was also considered as a co-regulatory factor of full-length SIRT1 [23].

So far, very few studies focus on the effects of aging and oxidative senescence on NK cells. The effects of calcitriol on SIRT1-ΔExon8 have not been reported as well. Therefore, this study aimed at investigating the effects of calcitriol on the aging and oxidative senescence of NK cells, as these cells occupy a central position in the human innate immune system.

## 2. Materials and methods

### 2.1 Cell culture and reagents

Three human peripheral blood samples were acquired from donors who did not have any active autoimmune disease, acute or chronic inflammatory disease, cancer, or other immune-related diseases. The age of the donors ranged from 48–65 years. Peripheral blood mononuclear cells (PBMCs) were isolated using Lymphoprep Ficoll. The NK cells were expanded and maintained using an NK Cell Culture Kit (MoreCell, Shenzhen, China) at 37°C/5% CO2.

### 2.2 Flow cytometric analysis

After treatment with calcitriol for 72 h, the NK cells were stained with anti-human FITC-CD3, PerCp-CD56, APC-CD16, PE-NKG2A, PE-TIM3, PE-PD1, and PE-KIR (BioLegend, California, USA). All the FACS assays were performed using DxFLEX Flow Cytometer (Beckman, California, USA).

### 2.3 Cell expansion measurement, cytokine-releasing and cell cycle assay

Cell expansion was determined using a Cell Counting Kit-8 [24] (CCK8; Beyotime, Shanghai, China). NK cells were treated with calcitriol for 48 h and the supernatant was collected for the Cytokine-releasing assay. The cytokine levels were examined using the LEGEND Plex Human Inflammation Panel (BioLegend, California, USA). First, the cytokine capture beads were incubated with the standards or samples and then further incubated with biotinylated detection antibodies. The biotinylated detection antibody-binding solution, streptavidin (SA)-PE, was subsequently added to provide the fluorescent signal [25]. These signals were then analyzed using the FACS assay.

Cell cycle was measured using a Cell Cycle Detection Kit (Beyotime, Shanghai, China). NK cells were treated with calcitriol for 72 h. These cells were fixed in 70% ice-cold ethanol and then dyed with ribonuclease A (RNase A) and propidium iodide (PI) (Beyotime, Shanghai, China) [26].

### 2.4 NK cell-killing assay

The human erythroleukemic cells, K562 cells (ATCC, Virginia, USA), were incubated with 10 μM 3,3′-dioctadecyl-oxacarbocyanine (DIO; Beyotime, Shanghai, China) for 15 min and then added to the calcitriol-treated NK cells at different ratios (E/T = 1:1, 5:1 and 10:1) for 4 h, respectively [27].

### 2.5 Oxidative senescence induction and X-gal staining analysis

The in vitro NK cell aging model using hydrogen peroxide (H2O2). In order to determine the anti-oxidation effect of calcitriol, NK cells were first treated with calcitriol and then exposed to 100 μM H2O2 for 24 h.

The activity of β-galactosidase was measured using 5-bromo-4-chloro-3-indolyl-β

-D-galactopyranoside (X-gal) staining. The treated cells were fixed in 4% paraformaldehyde and stained with β-galactosidase staining solution (Beyotime, Shanghai, China) overnight at 37°C [28]. The stained cells were then suspended in PBS and images of these cells were acquired using a Leica fluorescence inversion microscope system. Three microscopic images were randomly collected from different positions at 40× magnification for each group. The percentage of X-gal-stained cells was calculated and used to assess the aging of cells using the ImageJ software V.1.45S.

### 2.6 Staining and analysis of the mitochondrial membrane potential

Chloromethyl-X-Rosamine (CMXRos) and Hoechst (Beyotime, Shanghai, China) was used to assess the mitochondrial membrane potential. The cell nuclei (blue) were located by Hoechst staining, while the mitochondrial membrane potential was determined by the mitochondrial specificity-denoting fluorescent dye, CMXRos. The Hoechst+ CMXRos- cells (marked by white arrows) showed low activity. The relative fluorescence level was calculated by dividing the number of Hoechst positive cells by CMXRos-positive cells [29]. Cells were incubated with 200 nM CMXRos and Hoechst (30 min, 37°C) and detected using a Leica inverted microscope at excitation wavelengths of 579 nm and 350 nm. The percentage of Hoechst^+^ CMXRos^+^ cells was calculated as described in Section 2.5.

### 2.7 Western blotting

The cells were lysed using the radioimmunoprecipitation assay (RIPA) lysis buffer (Beyotime, Shanghai, China) with phosphatase inhibitor PhosSTOP (Roche, Basel, Switzerland) and 1 μM phenylmethylsulfonyl fluoride (PMSF) (Beyotime, Shanghai, China). Total protein was quantified using the Bradford Protein Assay Kit (TAKARA, Tokyo, Japan). The protein samples were separated by sodium dodecyl sulfate-polyacrylamide gel electrophoresis (SDS-PAGE) and transferred to 0.22 μm polyvinylidene fluoride (PVDF) membranes (Immobilon-P membrane; Millipore, Massachusetts, USA). After blocking, the membranes were probed with primary antibodies and incubated with the secondary antibodies [30]. Membranes were detected using the Odyssey system (LI-COR, Nebraska, USA).

### 2.8 Statistical analysis

The data was analyzed by one-way analysis of variance (ANOVA) using GraphPad Prism and presented as mean ± standard deviation (SD). Differences were considered statistically significant at P < 0.05. The results were also analyzed using the GraphPad software.

## 3. Results

This study focused on the anti-aging effects of calcitriol on NK cells. The results showed that Calcitriol decreased the expression of aging-related biomarkers including CD16, TIM3, PD-1, KIR and increased the expression of NKG2A of NK cells. it also inhibited NK cell proliferation and arrest them in G1 phase. Calcitriol decreased the releasing of inflammatory factors such as IL-5, IL-13, IFN-γ, TNF-α and had a certain antioxidation effects, which could contribute to its anti-aging effects.

### 3.1 Calcitriol reversed the expression of the surface aging markers, slowed down the NK cell amplification, and maintained NK cells in the G1 phase.

**Figure 1.**
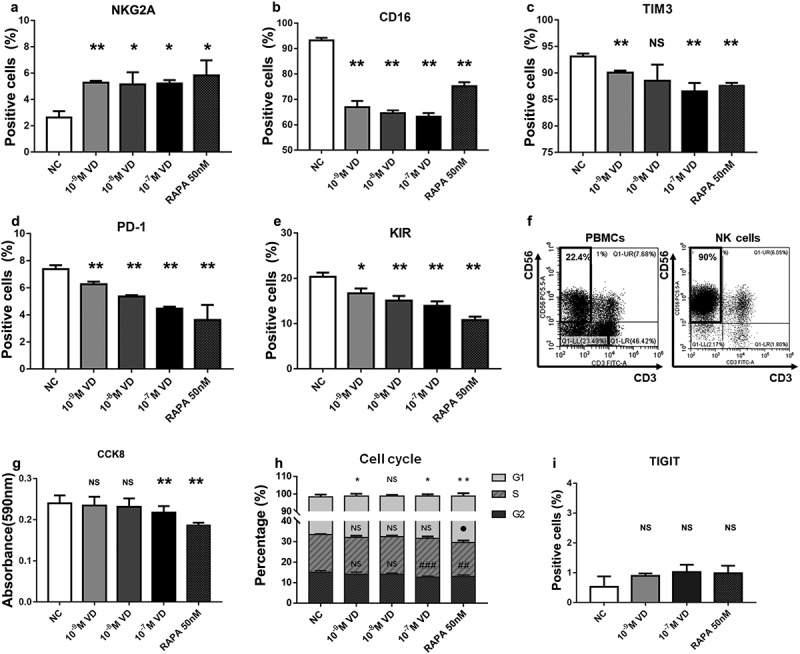
Aging-related phenotype, cell expansion, and cell cycle analysis of NK cells. (a-e) The human NK cells treated with calcitriol or rapamycin for 72 h showed the following cell biomarkers: natural killer group 2 member A (NKG2A), cluster of differentiation 16 (CD16), T cell immunoglobulin and mucin domain-containing protein 3 (TIM3) programmed cell death-1 (PD-1), and killer immunoglobulin-like receptor (KIR), which were related to aging. 50 nM rapamycin (RAPA) was used as the positive control. n = 3 for each group. *P < 0.05 and **P < 0.01 were compared with the negative control (NC) group. (f) NK cells (right) isolated from human peripheral blood mononuclear cells (PBMCs, left) by Magnetic bead sorting. (g) Cell proliferation analysis measured by cell counting kit 8 (CCK8). (h) Effect of calcitriol or rapamycin on the cell cycle in sorted NK cells after treatment for 72 h. *P < 0.05, ●P < 0.05, and #P < 0.05 indicated the significant differences in the G1, S, and G2 phases, respectively. All the groups were compared with NC, respectively. n = 3. (i) The percentage of T cell immunoglobulin and immunoreceptor tyrosine-based inhibitory motif (ITIM) domain (TIGIT)-positive NK cells after treatment with calcitriol or rapamycin for 72 h. *P < 0.05 and **P < 0.01 were compared with NC. n = 3

Senescent NK cells showed high expression of aging-related biomarkers, such as CD16, PD-1, TIM3, and KIR, and low expression of NKG2A. We found that the expression levels of the aging-related biomarkers NKG2A (Figure 1a) (*P < 0.05) increased, while the expression of CD16 (Figure 1b), TIM3 (Figure 1c), PD-1 (Figure 1d), and KIR (Figure 1e) (*P < 0.05) were decreased after treatment with calcitriol for 72 h. The reversal effects were positively correlated with the concentration of calcitriol (Supplementary Figure 1).

We expanded cells from the human peripheral blood samples in vitro. Then we isolated the NK cells (CD3-CD56+) by magnetic bead sorting (figure 1f). The sorted NK cells were then cultured with different concentrations of calcitriol or rapamycin for 72 h. CCK8 staining results showed that the growth rate of the calcitriol-treated NK cells of high concentration (10^−^[7] M) was slower than that of the negative control group (NC) (*P < 0.05) (Figure 1g). In addition, calcitriol regulated the NK cell cycle. After treatment with calcitriol for 48 h, NK cells showed a high calcitriol concentration-dependent shift toward the G1 phase (*P < 0.05), while the proportion of cells in the G2 phase (#P < 0.05) decreased. The proportion of calcitriol-treated NK cells in the S phase showed no change, regardless of the concentration of calcitriol (Figure 1h, Supplementary Figure 2). We then tested whether this calcitriol-derived G1 phase arrest induced NK cell apoptosis and found that the NK cell apoptotic biomarker, T cell immunoglobulin and immunoreceptor tyrosine-based inhibitory motif (ITIM) domain (TIGIT), showed no significant change (P > 0.05, NS) (Figure 1i). These results implied that addition of calcitriol reduced the growth rate of NK cells, decreased their proliferation, and induced G1 phase arrest of the NK cells instead of causing apoptosis.

### 3.2 Calcitriol decreased the release of inflammatory cytokines in NK cells and inhibited the cytotoxicity of NK cells

**Figure 2.**
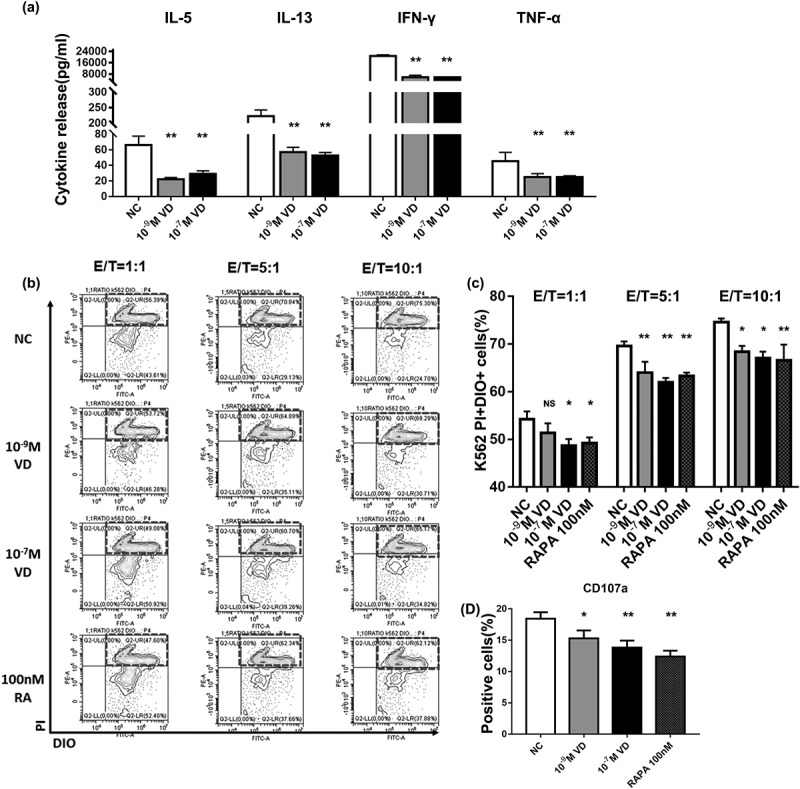
Effects of calcitriol on the functions of NK cells. (a) Analysis of the cytokine released by the calcitriol-treated NK cells in 48 h. (b) Fluorescence-activated cell sorting (FACS) results showed dead K562 cells labeled by 3,3′-dioctadecyl-oxacarbocyanine (DIO) and propidium iodide (PI). K562 cells were co-cultured with different ratios of calcitriol-treated NK cells for 4 h. (c) FACS analysis of dead K562 cells in NK-killing assays. (d) The degranulation effects of treated NK cells in killing assays. n = 3 for each group. *P < 0.05 and **P < 0.01 were compared with NC

Inflammageing is a chronic low-grade inflammation. It accelerates the aging of NK cells, as NK cells participate in this chronic inflammation. Figure 2a demonstrates that the levels of IL-5, IL-13, IFN-γ, and TNF-α significantly decreased in all groups than in the negative control (*P < 0.05). IFN-γ and TNF-α are well-known pro-inflammatory cytokines but some studies have also shown the pro-inflammatory properties of IL-5. IL-13 has been reported to contribute to different types of mucosal inflammation, such as allergic asthma, ulcerative colitis, eosinophilic esophagitis, and several diseases related to fibrosis.

However, the cytotoxicity of NK cells mainly involves the release of inflammatory cytokines and degranulation of cells. We labeled K562 cells with DIO and co-cultured the DIO-K562 cells with calcitriol-treated NK cells for 4 h. The percentage of DIO^+^PI^+^ cells were lower in all the effector (E)/target (T) ratio groups than in the negative control (*P < 0.05) (Figure 2b,c). The deduced expression of cluster of differentiation 107 (CD107) indicated lower degranulation (*P < 0.05) (Figure 2d). These results show the inhibition of the NK-killing function by calcitriol.

### 3.3 Calcitriol activated the expression of SIRT1-∆Exon8 and anti-aging-related SIRT1/pERK pathway

**Figure 3.**
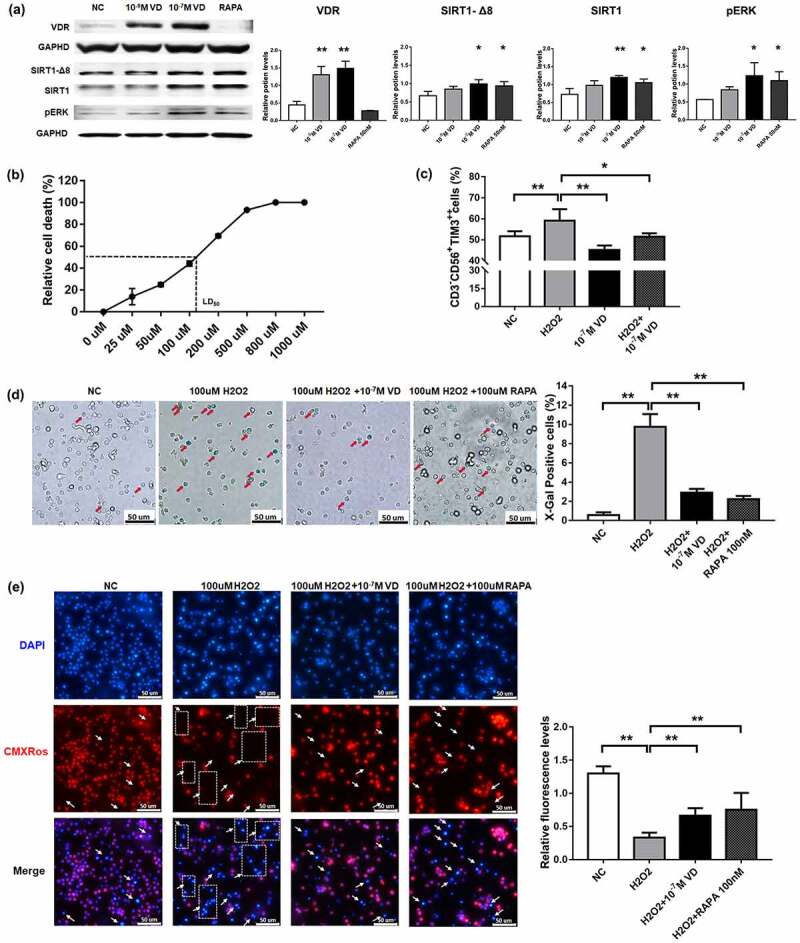
The anti-aging effects of calcitriol on NK cells. (a) The expression of the vitamin D receptor (VDR), sirtuin 1 (SIRT1), SIRT1-∆Exon8, and protein kinase R-like endoplasmic reticulum kinase (pERK) in NK cells was detected by western blotting analysis after treatment with calcitriol for 48 h. Glyceraldehyde-3-phosphate dehydrogenase (GAPDH) was used as the loading control. Bar diagram illustrating the western blotting results (n = 3). *P < 0.05 and **P < 0.01 were compared with NC (n = 3). (b) Dosage mortality curve of NK cells after treatment with hydrogen peroxide (H_2_O_2_) for 24 h. (c) Percentage of TIM3 cells exhibiting high expression after treatment with 100 μM H_2_O_2_ for 24 h. (d) The senescence-associated β-galactosidase staining and analysis results. The senescent cells were stained in blue and indicated by red arrows. Scale bar, 50 μm. (e) NK cells were stained with Hoechst and Chloromethyl-X-Rosamine (CMXRos) to determine the cell location and mitochondrial membrane potential. The aging and apoptotic cells were Hoechst^+^ CMXRos^−^ and indicated by white arrows. Scale bar, 50 μm. Statistical analysis showed relative fluorescence intensity. *P < 0.05 and **P < 0.01 were compared with H_2_O_2_ group in figures C, D, and E (n = 3)

To investigate the anti-aging effects of calcitriol on NK cells, we detected the aging-related signaling pathways by immunoblotting. Western blotting analysis was performed to assess the expression levels of VDR, SIRT1-∆Exon8, SIRT1, and PERK in the calcitriol-treated NK cells. Statistical analysis showed that calcitriol activated the VDR/SIRT1/pERK axis at high concentrations. Meanwhile, calcitriol activated the expression of SIRT1-∆Exon8 (*P < 0.05). (Figure 3a).

### 3.4 Calcitriol resisted the aging of NK cells induced by oxidative stress in vitro

It is widely accepted that aging is caused by oxidative damage accumulation due to the large body of evidence found over the years [31]. We cultured the NK cells with different concentrations of H_2_O_2_ for 24 h and found that 100 μM was the nearest concentration to the median lethal dose (LD_50_) (Figure 3b). The expression of TIM3 in the NK cell population increased by the addition of H_2_O_2_, while the expression decreased after treatment with calcitriol (*P < 0.05) (Figure 3c). In addition, aging NK cells were stained blue in the presence of β-galactosidase. This blue color of the cells is a specific hallmark of senescent cells. Statistical analysis demonstrated that untreated NK cells have less cellular β-galactosidase activity, while the NK cells exposed to H_2_O_2_ displayed elevated β-galactosidase activity. Calcitriol reduced the β-galactosidase-positive population, indicating the presence of fewer aging cells (*P < 0.05) (Figure 3d).

Decreased mitochondrial membrane potential has also been observed in a variety of aging cells from several mammalian species. We evaluated the mitochondrial membrane potential of the NK cells in this study. The statistical results showed that H_2_O_2_ significantly decreased the cellular activity of NK cells compared to that of the untreated cells, while calcitriol increased the damage caused by H_2_O_2_ (*P < 0.05) (Figure 3e).

### 3.5 Calcitriol resisted the apoptosis of NK cells induced by oxidative stress in vitro

**Figure 4.**
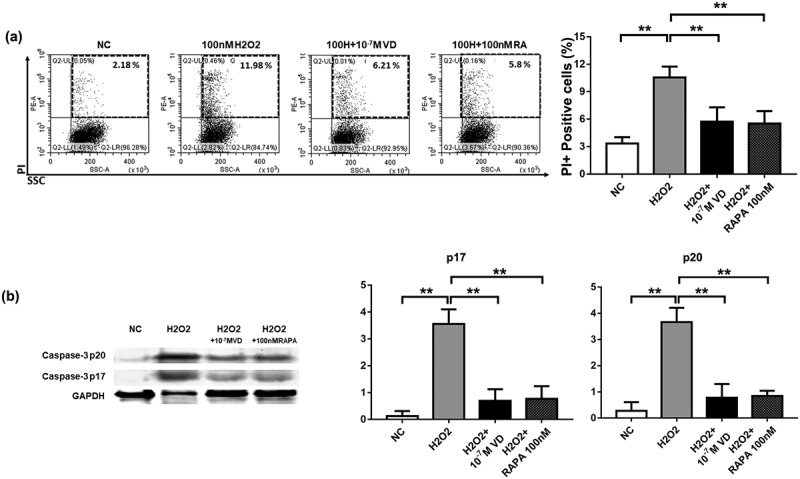
The anti-apoptotic effects of calcitriol on NK cells. (a) Anti-apoptotic effects of calcitriol in H_2_O_2_-induced injury. FACS analysis showed the percentage of apoptotic NK cells after treatment with 100 μM H_2_O_2_ for 24 h. (b) The expression levels of caspase-3 p20 and caspase-3 p17 in NK cells were detected by western blotting analysis after treatment with 100 μM H_2_O_2_ for 24 h. GAPDH was used as the loading control. Bar diagram illustrating the western blotting results (n = 3). *P < 0.05 was compared with the H_2_O_2_ group

Next, we measured the number of apoptotic cells. The flow cytometry results showed that treatment with 100 μM H_2_O_2_ for 24 h led to significant apoptosis of the NK cells. However, treatment with calcitriol decreased the apoptotic ratio (*P < 0.05) (Figure 4a).

All the above results demonstrated the resistance of calcitriol against oxidative stress caused by H_2_O_2_. However, we detected apoptotic proteins in NK cells under oxidative stress. Before treatment with H_2_O_2_, 10^−^[7] M calcitriol was added to the NK cells for 24 h. The levels of apoptosis-related proteins, caspase-3 p17 and caspase-3 p20, increased significantly after treatment with H_2_O_2_. Calcitriol decreased H_2_O_2_-derived apoptosis and downregulated the expression of caspase-3, p17, and caspase-3 p20 (*P < 0.05) (Figure 4b). These results indicated that calcitriol resisted apoptosis induced by H_2_O_2_.

## 4. Discussion

The long-term interaction between inflammation and oxidative stress is an important factor in aging. In addition, inflammation and oxidative stress are synergistic [32]. Excess ROS generated by oxidative stress attack the body cells. During this process, inflammatory factors are released by the activated immune system to kill the abnormal cells, which leads to further ROS generation. Lack of calcitriol can cause autoimmune diseases, chronic metabolic diseases, and tumors. However, little is known about the anti-aging effects of calcitriol on the immune system, especially on NK cells.

In this study, we investigated the anti-aging effects of calcitriol on NK cells. We found that calcitriol increased the expression of NKG2A, while decreasing the expression of KIR *in vitro*. The CD56^−^CD16^+^ subpopulation of NK cells, which is known to be related to chronic inflammation, is significantly increased in the elderly [33]. Therefore, the reduction in CD16 expression induced by calcitriol might be attributed to its anti-inflammatory effect. TIM-3 and PD-1 have been reported to be the inhibitory and depletion receptors of NK cells. Our study showed that calcitriol downregulated the expression levels of TIM-3 and PD-1 and prevented apoptosis of NK cells.

In addition, calcitriol inhibited the expansion of NK cells and maintained the NK cell population in G1 phase *in vitro*. However, the cells in the S-phase were not affected. TIGIT, which is known as a checkpoint factor of NK cells, remained unchanged. These results indicated that calcitriol did not lead to the death of NK cells, but it only slowed down the rate of NK cell expansion.

It’s well known that inflammation leads to immunosenescence [34,35]. Furthermore, we found that calcitriol reduced the levels of IL-5, IL-13, IFN-γ, and TNF-α in NK cells. The cytotoxicity of NK cells is partly mediated by the release of inflammatory factors [36]. Moreover, there was a decrease in the mortality of K562 tumor cells and degranulation of NK cells. These results were in line with our expectations. Rapamycin is a well-known drug that reduces the metabolic rate to prevent aging[37] which used as the positive control in this study. It results in immunosuppression and increases the potential risk of tumor and tumor burden. This burden has been confirmed in mouse models of breast cancer [38]. Whether calcitriol exhibits similar effects to rapamycin remains unknown. More animal and clinical studies should be performed to verify their effects in the human body.

Finally, we studied the effects of calcitriol on antioxidant senescence. Resveratrol, a well-known anti-aging drug, can downregulate the NF-κB pathway by activating the SIRT1/PERK axis. This downregulation has also been shown to be beneficial in neurodegenerative disorders and cardiovascular diseases [39]. SIRT1 deacetylation is associated with metabolic control and mitochondrial biogenesis. In this type of regulation, elevated SIRT1 levels can promote the accumulation of peroxisome proliferator-activated receptor gamma coactivator-1 alpha (PGC-1α) in the nucleus, which results in the transcription of genes that are necessary for mitochondrial function [40]. Besides, SIRT1 suppresses the mammalian target of rapamycin (mTOR) signaling pathway, which can protect the nerves and resist brain aging in mice [41]. In this study, we identified a similar regulatory pathway for calcitriol. Calcitriol activated SIRT1 via VDR and raised the SIRT1/pERK axis. In addition, we found calcitriol upregulated the expression of SIRT1-∆Exon8. Stresses are known as an up-regulatory factor of SIRT1-∆Exon8. Although SIRT1-∆Exon8 itself displays reduced p53 deacetylation activity, it exerts an additive deacetylation effect on p53 when expressed together with full‐length SIRT1 [42]. This effect indicated the anti-apoptotic function of calcitriol on NK cells. However, the role of SIRT1-∆Exon8 in age-related pathways remains to be better understood.

Since the decline of SIRT1 might be due to oxidative damage [43], we established an acutely aging NK cell model *in vitro* using H_2_O_2_. The oxidative aging induced by H_2_O_2_ increased the expression of TIM3 as well as the activity of β-galactosidase in NK cells. We found that calcitriol resisted the oxidative damage and maintained the mitochondrial activity and cell viability of NK cells, while preventing cell apoptosis. However, calcitriol has not yet been identified as an anti-aging drug in clinical medicine as more evidence is needed regarding the anti-aging effects of this drug *in vivo*. In summary, we provided preliminary evidence to implicate the anti-aging effects of calcitriol on NK cells *in vitro*. We believe that the effects of this drug should be further explored in future animal and clinical studies.

## 5. Conclusion

In this study, we demonstrated the anti-aging effects of calcitriol on NK cells. Calcitriol reversed the expression of aging biomarkers on NK cells. It slowed down NK amplification and kept them in G1 phase. In addition, calcitriol inhibited the release of inflammatory cytokines and down-regulated degranulation of NK cells. Activation of SIRT1-∆Exon8 and VDR/ SIRT1/pERK axis by calcitriol might be the main mechanism of calcitriol anti-aging effect based on all above. Finally, calcitriol resisted the oxidative senescence of NK cells in vitro. Further studies should focus on animal and clinical research.

## Supplementary Material

Supplemental Material

## Highlights

1. Calcitriol decreased the expression of ageing-related biomarkers including CD16, TIM3, PD-1, KIR and increased the expression of NKG2A of NK cells.
2. Calcitriol was able to inhibit NK cell proliferation and arrest them in G1 phase.
3. Calcitriol decreased the releasing of major inflammatory factors such as IL-5, IL-13, IFN-γ and TNF-α of NK cells.
4. The anti-ageing effect of calcitriol on NK cells can be achieved through the Sirt1-pERK signal pathway.
