# Supplementary material for: Study of calcitriol anti-aging effects on human natural killer cells in vitro
Source: Bioengineered. 2021 Sep 21;12(1):6844–54. doi: 10.1080/21655979.2021.1972076 (PMC8806577; doi:10.1080/21655979.2021.1972076)
Supplement: Supplemental Material [file KBIE_A_1972076_SM2800.zip › supplementary/LEGENDS.docx]

Supplementary Fig.1:

Flow cytometric analysis of the NK cells ageing phenotype.

Figures (left) were obtained by gating the CD3^-^CD56^+^ cells.

Supplementary Fig.2:

Cell cycle analysis of sorted NK cells after treatment with calcitriol for 72 h.
