# Supplementary figures and images for: Study of calcitriol anti-aging effects on human natural killer cells in vitro
Source: Bioengineered. 2021 Sep 21;12(1):6844–54. doi: 10.1080/21655979.2021.1972076 (PMC8806577; doi:10.1080/21655979.2021.1972076)

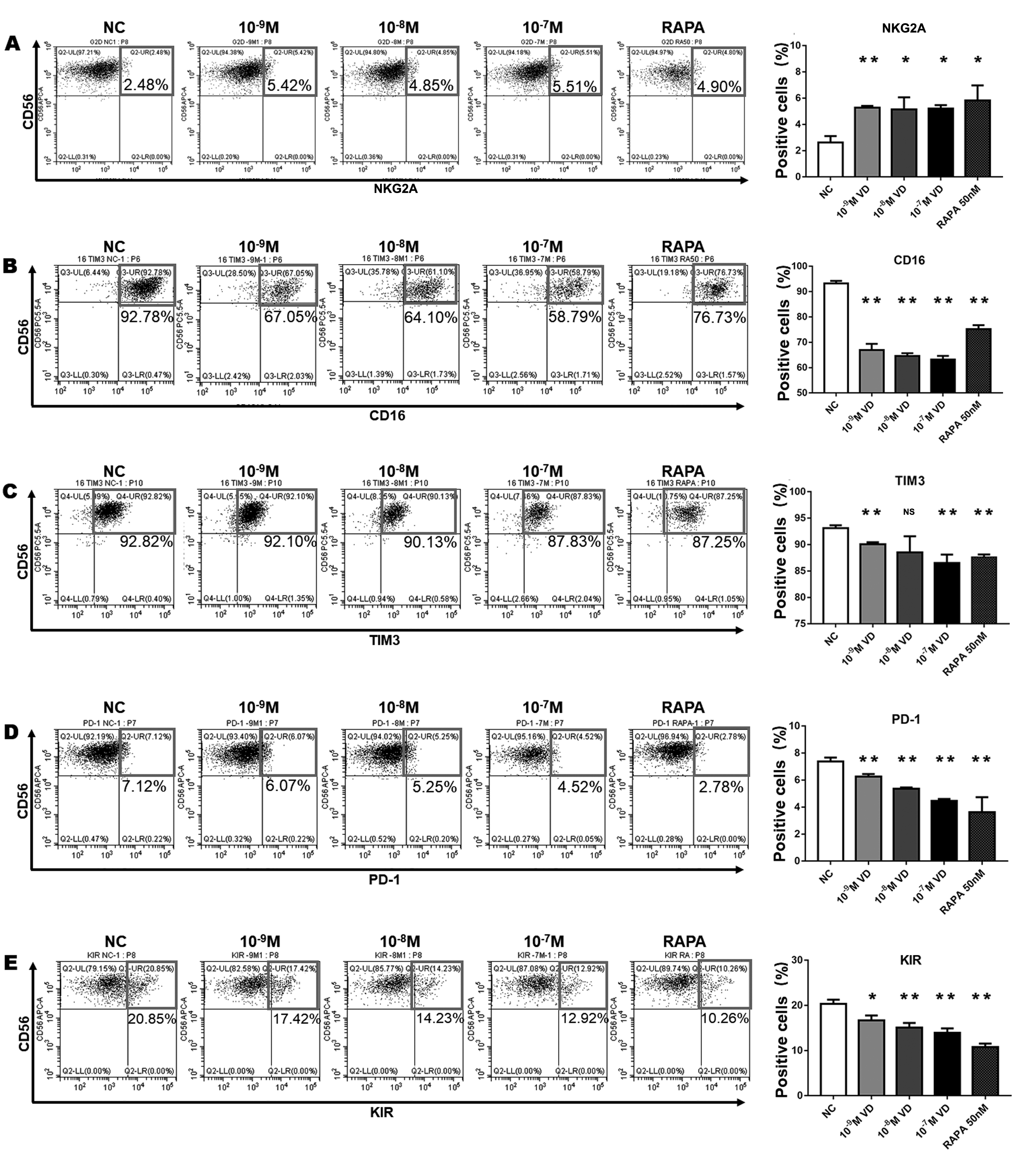

Supplement: Supplemental Material [file KBIE_A_1972076_SM2800.zip › supplementary/Supplementary FIG 1.tif]

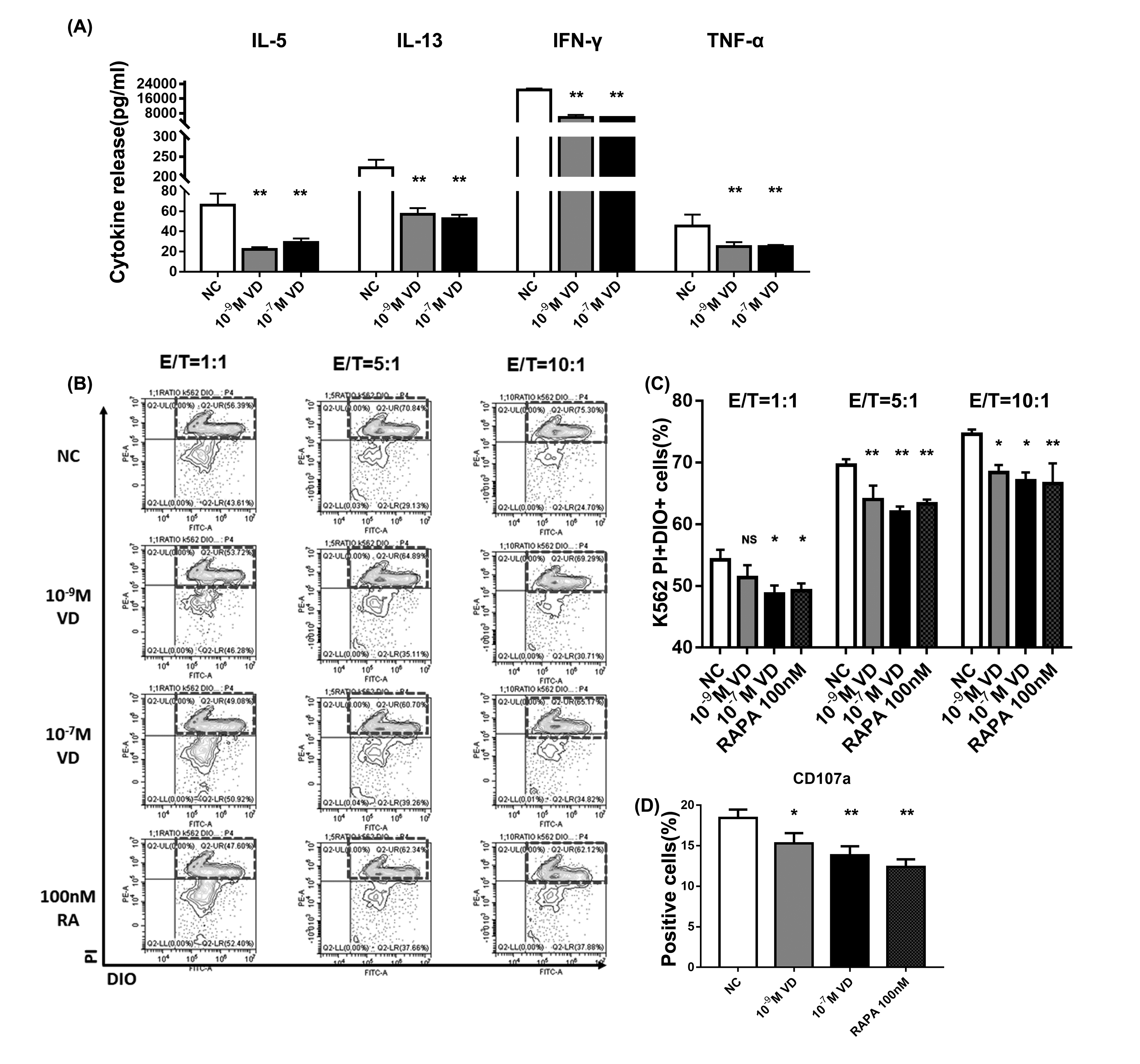

Supplement: Supplemental Material [file KBIE_A_1972076_SM2800.zip › supplementary/supplementary FIG2.tif]
